# Winner of the Ronald Melzack – *Canadian Journal of Pain* 2021 Paper of the Year Award / Récipiendaire du Prix Ronald Melzack Pour L’Annee 2021 Des Articles Parus Dans *La Revue Canadienne de La Douleur*

**DOI:** 10.1080/24740527.2022.2094756

**Published:** 2022-07-20

**Authors:** Joel Katz, Anna Waisman

**Affiliations:** Canadian Journal of Pain

Dr. Lise Dassieu and colleagues Drs. Angela Heino, Élise Develay, Jean-Luc Kaboré, M. Gabrielle Pagé, Gregg Moor, Maria Hudspith, and Manon Choinière are the winners of the Ronald Melzack–*Canadian Journal of Pain* 2021 Paper of the Year Award for their article titled “‘They think you’re trying to get the drug’: Qualitative investigation of chronic pain patients’ health care experiences during the opioid overdose epidemic in Canada.”^1^ Dr. Dassieu received the accolade from Dr. Loren Martin, who, on behalf of Dr. Lynn Gauthier, chair of the Canadian Pain Society’s (CPS) Awards Committee, announced the winning article at the Annual Scientific Meeting of the CPS in Montréal, Quebec—the first face-to-face CPS meeting since the COVID-19 pandemic began. The winning paper was selected from all original articles published in the *Journal* in 2021 and was determined by the Editor-in-Chief based on rankings from the *Journal* Editorial Board members, who considered originality, novelty, quality, and potential impact when submitting their votes.

The winning article documents the ongoing harms done to Canadians with chronic pain in the wake of the 2017 Canadian “Guideline for Opioid Therapy and Chronic Non-Cancer Pain,”^2^ which introduced strict dose limits for people taking opioids, fostered a climate of fear among prescribers, and opened a Pandora’s box of anguish and helplessness among patients. Dassieu and colleagues conducted a qualitative study examining the impact of the opioid epidemic on 22 individuals living with chronic pain, who, in 2019, were taking opioids for pain, had taken them in the past, or had never used them. The authors recruited participants from two Canadian provinces known to have been differentially affected by the opioid crisis, namely, British Columbia and Québec. During the interview, participants provided information on various topics, including their relationships with health care providers and their opinions on recent changes to prescription policies and media coverage regarding opioids. The authors analyzed the data using a thematic analysis framework that identified common themes between participant narratives.

The results of the study revealed five key themes supported by poignant and moving verbatim quotations from participants. First, participants reported being urged to taper their use of opioids, often at the cost of patients’ autonomy, personal needs, and ability to function at the lower dose. Next, participants reported being stigmatized and subjected to discrimination by health care providers; for example, being labeled “addicts,” accused of drug-seeking behavior, and denied treatment. In some cases, individuals even reported forgoing opioid medication to win their provider’s trust and avoid stigmatization, despite the negative consequences this had on their pain and lives more generally. However, another prominent theme revealed that the strength of the patient–provider relationship protected against much of this discrimination. Relationships in which providers knew their patients and had long-standing relationships with them were associated with better access to pain medication and fewer damaging interactions. In addition, differences in individuals’ abilities to self-advocate, which largely depended on their capacity to understand and communicate with providers using medical terminology, determined the extent to which they felt equipped to protect themselves against the addict stigma and obtain the treatment they sought. Finally, the study findings revealed participants’ general tendency to differentiate and distance themselves from those they perceived as addicts.

One of the greatest contributions of the study is giving individuals living with chronic pain a voice to express their perspectives and experiences in the context of the opioid crisis. The findings highlight how prejudices within the health care system present far-reaching, daily consequences for individuals’ pain management and quality of life. Though efforts to reduce unsafe prescribing of medications are important, this study underscores the damaging impact that strict implementation of prescription guidelines has had on the lives of people living with chronic pain. Indeed, given the subjective and multidimensional facets of pain, treatment necessitates a compassionate, person-centered approach. Providers would do well to collaborate with their patients in making decisions, actively listen to their needs and priorities, and demonstrate openness and respectful consideration for patients’ unique histories. As pointed out by Dassieu et al., a biopsychosocial model of care involving a multidisciplinary team of professionals with specialized knowledge and skills should be encouraged in addressing individuals’ functional and psychological needs in addition to the medical dimensions of their health. Without such compassionate care and without appropriate medical access to opioids, individuals who live with chronic pain will seek opioids through alternative, illicit means.^3^

Dassieu and colleagues also illustrate how discrimination and bias permeate all levels of the health care system, from physicians, nurses, and pharmacists to the public at large. At the provider level, structural inequities were found to impact participants in diverse ways, in part depending on their health literacy. As part of fostering their client-centeredness, providers require appropriate training to inform them about social inequities associated with chronic pain^4^ and help them develop cultural competence to advocate change and better meet their clients’ needs.^5^ It is crucial that health care providers develop appropriate communication and educational skills that acknowledge and respect others’ views, including minimizing use of medical jargon during the patient encounter.^6^ Finally, as Dassieu at al. note, the desire of participants to differentiate themselves from “addicts” points to another layer of prejudice, this one among people with chronic pain who are taking opioids. One of the explanations offered by the authors for this finding concerns the generally negative way in which opioids are represented in the media. Public coverage of the opioid epidemic could provide a more balanced portrayal of opioid use in people with chronic pain and make an effort to destigmatize the legitimate use of opioids for chronic pain.^7^

In the span of a few years, the landscape has begun to change for the better for most people taking opioids for chronic pain. The backlash^8–10^ that occurred in response to the 2016 Centers for Disease Control and Prevention (CDC) Guideline for Prescribing Opioids for Chronic Pain and the 2017 Canadian “Guideline for Opioid Therapy and Chronic Non-Cancer Pain”^2^ has resulted in a sober second look at the damage done to people taking opioids for chronic pain. The CDC has issued an updated Draft CDC Clinical Practice Guideline for Prescribing Opioids^11^ that removes dose limits, emphasizes flexibility, and encourages good clinical judgment when prescribing. Presumably, Canada will follow suit. Nevertheless, despite the promise of change, people with chronic pain still struggle to find physicians willing to prescribe opioids and providers open to helping them manage their pain.^12,13^

The Editorial Board and the CPS Awards Committee offer our most heartfelt congratulations and thanks to Dr. Dassieu and colleagues for their award-winning article and for choosing to publish in the *Canadian Journal of Pain*. We look forward to next year’s CPS meeting in Banff, Alberta, where we plan to announce two award winners: one for the basic/preclinical sciences and another for the applied/clinical sciences. Please submit your original research to the *Canadian Journal of Pain* and become eligible for The Ronald Melzack–*Canadian Journal of Pain* 2022 Paper of the Year Award/Prix Ronald Melzack Pour L’Annee 2022 Des Articles Parus Dans *La Revue Canadienne De La Douleur*.
